# 

**Published:** 1887-01

**Authors:** 


EDITOR:

S. J. JONES, M. D. LL. D.

COLLABORATORS.

CHICAGO:

PROFESSOR E. ANDREWS, M.D., LL.D.
PROFESSOR J. BARTLETT, M.D.
PROFESSOR W. H. BYFORD, A.M., M.D.
PROFESSOR L. CURTIS, A.M., M.D.
PROFESSOR O. C. DEWOLF, A.M., M.D.
PROFESSOR E. C. DUDLEY, A.B., M.D.
PROFESSOR C. FENGER, M.D.
PROFESSOR E. WING, A.M., M. D.
PROFESSOR J. N. HYDE, A.M., M.D.
PROFESSOR E. F. INGALS, A.M., M D.
PROFESSOR W. W. JAGGARD, A.M., M.D.
PROFESSOR F. S. JOHNSON, A.M., M.D.
PROFESSOR H. A. JOHNSON, M.D., LL.D.
PROFESSOR C. W. PURDY, M.D.
PROFESSOR C. G. SMITH, A.M., M.D.
PROFESSOR F. BILLINGS, M.D.
PROFESSOR W. T. BELFIELD, M.D.

MILWAUKEE, WISCONSIN:

PROFESSOR N. SENN, M.D.

WASHINGTON, D. C.:

S. O. RICHEY, M.D.

UNITED STATES ARMY:

SURGEON D. L. HUNTINGTON, M.D.

UNITED STATES NAVY

MEDICAL DIRECTOR J. M. BROWNE, M.D.
MEDICAL DIRECTOR A. L. GIHON, A.M., M.D.

### REPORTS AND PAMPHLETS RECEIVED.

---

Annual Report of the Commissioner of Pensions for the Year Ending June 30, 1886.

Eighth Annual Report of the State Board of Health of Illinois.

Transactions of the Eighteenth Annual Session of the Texas State Medical Association.

Transactions of the American Otological Society, Nineteenth Annual Meeting.

Transactions of the Medical Association of the State of Missouri, Twenty-ninth Annual Session.

Transactions of the State Medical Society of Wisconsin, 1886.

Transactions of the Illinois State Medical Society, 1886.

Inter-State Notification: Its Principles as Demonstrated in the History of Yellow Fever at Biloxi, Mississippi.

*T. D. Crothers, M.D.* Certain Hereditary and Psychical Phenomena in Inebriety.

*L. Webster Fox, M.D., and George M. Gould, A.B.* The Human Color-Sense Considered as the Organic Response to Natural Stimuli.

*Charles W. Dulles, M.D.* The Mechanism of Indirect Fractures of the Skull.

*H. M. Bannister, M.D.* On Consciousness in Epilepsy.

*H. M. Bannister, MD.* On the Classification of Insanity in Asylums or Hospitals for the Insane.

*Robert Battey, M.D.* Antisepsis in Ovariotomy and Battey's Operation.

*Frank Billings, M.D.* Notes on Hospital Practice in Vienna.

*Robert J. Lee, M.D., F.R.C.P.* Three Lectures delivered at the Hospital for Sick Children, Great Ormond Street. I and II. On the Transmission of Syphilis. III. On the Earliest Record of Whooping-cough.

*W. H. Daly, M.D.* I. Laryngology and Its Cognate Branches in America. II. The Simplest and Most Efficient Treatment of Diphtheria.

*Joseph Edcil Winters, M.D.* The Relative Influences of Maternal and Wet-Nursing on Mother and Child.

*Joseph Holt, M.D.* Relation of Quarantine to Shipping Interests.

*Oscar J. Coskery, M.D.* Three Cases of Fracture of the Skull.

*C. H. Hughes, M.D.* Meconeuropathia.

*B. Bernard Browne, M.D.* The Curette as a Diagnostic and Therapeutic Agent in Gynecology and Obstetrics.

*Dr. J. Pirnat.* Diphtheria and Its Treatment.

SUBSCRIPTION PRICE, $3.00 PER YEAR, IN ADVANCE.

Whole Number, 325 JANUARY, 1887. Vol. LIV. Number 1.

LIBRARY INDEXED S.G.O.

INDEX MEDICUS

165

### THE CHICAGO MEDICAL JOURNAL AND EXAMINER

Editor: S. J. JONES, M.D., LL.D.

COLLABORATORS.

CHICAGO:

PROFESSOR E. ANDREWS, M.D., LL.D.
PROFESSOR JOHN BARTLETT, M.D.
PROFESSOR W. H. BYFORD, A.M., M.D.
PROFESSOR LESTER CURTIS, A.M., M.D.
PROFESSOR O. C. DEWOLF, A.M., M.D.
PROFESSOR E. C. DUDLEY, A.B., M.D.
PROFESSOR C. FENGER, M.D.
PROFESSOR J. N. HYDE, A.M., M.D.
PROFESSOR E. F. INGALS, A.M., M D.
PROFESSOR W. W. JAGGARD, A.M., M.D.
PROFESSOR F. S. JOHNSON, A.M., M.D.
PROFESSOR H. A. JOHNSON, M.D., LL.D.
PROFESSOR C. W. PURDY, M.D.
PROFESSOR CHAS. G. SMITH, A.M., M.D.

MILWAUKEE, WISCONSIN:
PROFESSOR N. SENN, M.D.

WASHINGTON, D. C.:
S. O. RICHEY, M.D.

UNITED STATES ARMY:
SURGEON, D. L. HUNTINGTON, M.D.

UNITED STATES NAVY:
MEDICAL DIRECTOR, J. M. BROWNE, M.D.
MEDICAL DIRECTOR, A. L. GIHON, A.M., M.D.

